# Active Inference Modeling of Socially Shared Cognition in Virtual Reality

**DOI:** 10.3390/s26020604

**Published:** 2026-01-16

**Authors:** Yoshiko Arima, Mahiro Okada

**Affiliations:** Center for Social and Psychological Research of Metaverse, Kyoto University of Advanced Science, Kyoto 615-8577, Japan

**Keywords:** active inference, virtual reality, human–robot interaction, eye movement synchrony, collaborative learning, socially shared cognition, gaze tracking

## Abstract

This study proposes a process model for sharing ambiguous category concepts in virtual reality (VR) using an active inference framework. The model executes a dual-layer Bayesian update after observing both self and partner actions and predicts actions that minimize free energy. To incorporate agreement-seeking with others into active inference, we added disagreement in category judgments as a risk term in the free energy, weighted by gaze synchrony measured using Dynamic Time Warping (DTW), which is assumed to reflect joint attention. To validate the model, an object classification task in VR including ambiguous items was created. The experiment was conducted first under a bot avatar condition, in which ambiguous category judgments were always incorrect, and then under a human–human pair condition. This design allowed verification of the collaborative learning process by which human pairs reached agreement from the same degree of ambiguity. Analysis of experimental data from 14 participants showed that the model achieved high prediction accuracy for observed values as learning progressed. Introducing gaze synchrony weighting ($\gamma_{0}\geq 0.5$) further improved prediction accuracy, yielding optimal performance. This approach provides a new framework for modeling socially shared cognition using active inference in human–robot interaction contexts.

## 1. Introduction

How can people share ambiguous concepts with others? This is a fundamental question in social cognition research and is related to applications in human–robot interaction (HRI). Recent advances in physical AI and embodied artificial intelligence are bringing humanoid robots into homes and workplaces, making human–robot collaborative interaction an imminent reality. However, to achieve effective human–robot collaboration in these settings, robots must master capabilities that humans execute effortlessly: coordinated behavior, sharing of ambiguous concepts, and rapid mutual adaptation during interaction. Despite remarkable progress in motion generation and imitation learning, the fundamental mechanisms underlying these collaborative processes—particularly how humans and artificial agents can establish shared understanding through embodied multimodal interaction—remain poorly understood.

Therefore, this research is positioned at the intersection of applied VR/HRI studies and embodied AI, focusing on multimodal integration of gaze sensing, motion sensing, and cognitive process modeling. By examining how pairs share ambiguous category concepts through gaze and touch behavior in virtual reality environments, we aim to provide foundational insights for designing socially intelligent robots capable of genuine collaborative interaction with humans.

Virtual reality (VR) research has enabled the study of joint attention (JA), including body movements and gaze behaviors in social interactions [1]. In the present study, we introduced a joint action task in which participants were paired with a bot avatar as their partner. Joint action in VR spaces can be understood through the framework of dynamic coupling, which extends beyond mere mirroring or alignment in joint action neuroscience to encompass complementary behaviors, mutual adaptation, and synergies [2]. Joint attention (JA) serves as the foundation for this coordinated movement. For example, in observational studies of joint improvised performances, velocity-based synchrony shapes judgments of coordination and aesthetic experience [3]. JA is supported by a whole-brain system that integrates triadic information processing concerning (a) self attention/behavior, (b) others’ attention/behavior in social interactions, and (c) common referents in space. This capacity to adopt a common perspective with others [4] underlies the formation of socially shared cognition. In this study, we quantify gaze synchrony and gaze object sequence (GOS) using Dynamic Time Warping (DTW) and integrate it into the active inference framework as a collaborative weighting parameter (γ) applied to the risk term, thereby examining how lower-level embodied synchrony contributes to higher-level socially shared cognition formation. The use of DTW as a synchrony parameter has also been demonstrated to be useful in human–robot collaboration (HRC) research [5].

The aim of this study is, from a psychological perspective, to deepen the understanding of how socially shared cognition is achieved. From an engineering perspective, the goal is to provide insights into the design of socially intelligent agents by modeling how symbol sharing, grounded in the body and objects, can be established. To achieve these aims, we attempted to model the process of concept sharing using active inference modeling, which provides a unified framework in cognitive neuroscience.

The primary objective of this study is to model, within an active inference framework, the process by which human–human pairs achieve shared category concepts in Session 4 after task ambiguity has been systematically controlled in Session 3. In Session 3, each participant is paired with a bot that is programmed to provide consistently incorrect feedback on a predefined subset of items, thereby intentionally manipulating the level of category ambiguity experienced by the participants.

Although differences between Sessions 3 and 4 are also explored in terms of gaze synchrony between bot–human and human–human pairs, the main purpose of Session 3 is to establish a controlled ambiguous context for category judgment. For this reason, the order of Session 3 and Session 4 is fixed in the experimental design.

### Interpersonal Synchrony and Socially Shared Cognition

In this study, to examine how category recognition is shared, we implemented a handle effect task [6] in a VR environment. The handle effect is a psychological experimental task where response times change depending on handle direction, similar to the Simon effect. Since this effect will be reported separately, we do not report it in this paper. Regarding previous studies relevant to this report, when such tasks are performed as collaborative work with divided roles, it has been suggested that joint action is influenced by whether participants share representations with others and whether they perceive their partner as an intentional agent [7,8].

However, such representation sharing hypotheses tend not to be supported. This is because individual cognitive processes are closed systems, and there is a lack of methods to demonstrate having the same representations as others. Therefore, in this study, while assuming that cognitive processes are internally closed, we examine them using active inference models that adapt to the environment. Active inference based on the free energy principle provides the following computational framework for modeling predictive learning processes [9,10,11]. Biological agents observe environmental changes resulting from active behaviors and update internal models about themselves and their environment through Bayesian inference [12]. The probability distributions of responses to possible actions are calculated as Kullback–Leibler (KL) divergence, and it is assumed that actions are selected to minimize prediction errors. In social contexts, active inference extends to modeling others’ mental states through mutual prediction, where synchronization emerges when agents share common generative models [13,14].

In human face-to-face communication, there is an unconscious process by which attention and behavior become spontaneously synchronized with those of others. A meta-analysis of studies on synchrony has shown that sensory and behavioral synchrony enhances prosocial attitudes and behaviors [15,16]. Studies have also demonstrated that behavioral synchrony influences conformity and social decision-making processes [17], while negative emotions can reduce interpersonal synchrony [18]. Interpersonal synchrony can also serve as an indicator of perceiving the other person as part of the self [19], and manipulating gaze direction in a categorization task has been reported to alter trust-related behaviors [20]. Even in virtual environments, avatars can produce synchrony effects similar to those observed in face-to-face interactions [21].

Our previous study [22] analyzed gaze and motor synchrony between human or bot avatar pairs as indicators of interpersonal coordination in VR handle tasks. The results suggested that the synchrony index, measured using Dynamic Time Warping (DTW), was associated with changes in behavior.

This study examines collaborative learning processes where pairs attempt to share ambiguous category concepts through body movements and gaze in virtual environments. In this study’s model, we extend the active inference framework to model collaborative decision-making by performing dual-layer Bayesian updates in predicting partner behavior and selecting self-actions. Furthermore, by integrating Dynamic Time Warping (DTW) into the active inference framework as a collaborative weighting parameter (γ) applied to the risk term, we examine how lower-level embodied synchrony contributes to higher-level socially shared cognition formation. We calculated the prediction accuracy of this model from actual measured values at each trial and examined the time-series changes as the learning process of the model.

A key theoretical contribution of this study is the extension of active inference to model socially shared cognition through dual-layer Bayesian inference, where individuals predict not only their partners’ actions but also iteratively update those predictions through social interaction. By validating this framework against empirical VR data, we demonstrate that this theoretical extension successfully captures collaborative convergence processes in ambiguous category learning.

## 2. Method

### 2.1. Participants

Fourteen undergraduate and graduate students participated in the experiment. Participants consisted of 14 university students (mean age = 20.43 years, SD = 0.50), including 9 females and 5 males. All participants had normal or corrected-to-normal vision. Participants’ prior experience with virtual reality was not formally controlled. However, the recruitment criteria specified that participants should have no prior experience participating in VR experiments. Each participant completed four sessions in a fixed order: Session 1 (Practice), Session 2 (Individual), Session 3 (Bot Pair condition), and Session 4 (Human Pair condition). We report analyses from Sessions 3 and 4, where participants engaged in collaborative categorization tasks. In Session 3, each of the 14 participants was paired with a bot avatar, creating 14 human-avatar pairs. All bot avatars used a neutral VRoid humanoid model with identical appearance and behavioral policies across all participants. In Session 4 (Human condition), participants were paired into 7 human-human pairs according to a predefined protocol based on consecutive participant IDs (e.g., IDs 1 and 2, 3 and 4). Pre-existing relationships between participants were not considered in the pairing protocol. The primary objective of this study is to examine whether active inference models can capture the shared cognition formation process in human pairs during Session 4. Category ambiguity was controlled by having the Bot provide incorrect responses for half of the objects in Session 3.

Role assignments were as follows: Host participants (odd-numbered IDs, assigned as peer-to-peer communication hosts) were instructed to touch only “Kitchen” category objects in the Bot Pair condition and only “Garage” category objects in the Human Pair condition. Client participants (even-numbered IDs) were instructed to touch only “Garage” category objects in the Bot Pair condition and only “Kitchen” category objects in the Human Pair condition.

The experiment procedure was approved by the Ethics Committee of the Kyoto University of Advanced Science (project no. 22H07). All methods were carried out in accordance with relevant guidelines and regulations. Twenty individuals were provided with written documentation explaining the experimental procedures and potential risks. Fourteen participants who signed the informed consent form participated in the experiment.

### 2.2. Task and Procedure

The VR Handle task required participants to judge whether the presented object belonged to their assigned category and touch it only if it did. Each trial began with a pre-object waiting phase (approximately 1.5 s total), followed by a fixation cross (“+”), and then object presentation for up to 3 s. Participants responded by moving their virtual hand toward the object to “touch” it. Only the first touch was recorded; simultaneous touches were not possible.

In the Bot Pair condition, the bot’s movements traced pre-recorded human motion data, while Easy and Hard categorization objects appeared randomly, the bot always responded correctly to Easy objects but always responded incorrectly to Hard objects. Specifically, for Hard objects, the bot did not touch objects belonging to its assigned category but did touch objects not belonging to its assigned category. Bot gaze behavior is described in Section 2.8.

### 2.3. Apparatus and Stimuli

The VR systems were established in two separate rooms to accommodate paired participants. Each system comprised an HTC VIVE Pro Eye HMD, two VIVE Controllers (2018), two SteamVR Base Station 2.0 units, and a computer. The VR environment was created using Unity (version 2021.3.1f1) with a server-client network architecture implemented via Netcode for Game Objects. In this environment, paired participants entered the same virtual space and interacted via physical actions; no audio communication was available.

**Figure 1:**
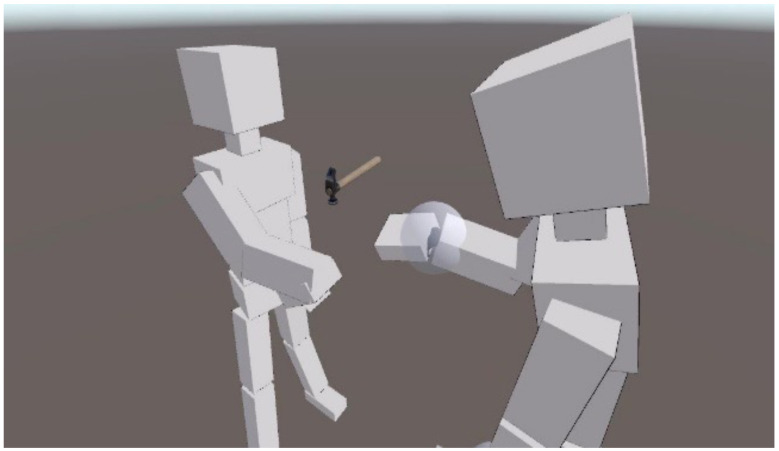
An example of task setting of the Joint Handle Task in VR space. *Note:* Due to image copyright issues, the avatars in this photograph have been replaced with boxes. In the actual experiment, neutral humanoid avatars were used, with gaze direction controlled through VRM LookAtHead components for bot avatars or real-time eye-tracking for human participants.

**Figure 2:**
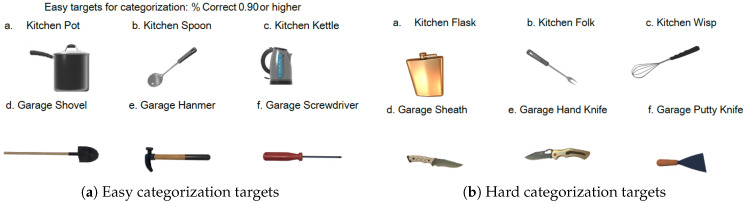
Categorization targets used in the experiment. Objects represent household items categorized as either “Kitchen” or “Garage” items, each with left-handle or right-handle orientation. (**a**) Easy categorization targets with accuracy rates of 0.90 or higher in preliminary testing (e.g., frying pan for Kitchen, drill for Garage). (**b**) Hard categorization targets with accuracy rates below 0.80, including ambiguous camping equipment requiring collaborative judgment between partners (e.g., portable stove, camping lantern). In the VR task, participants touched objects belonging to their assigned category. Objects were created using assets from Frogbytes [23], Yagunov [24], and High Quality Assets for Video Games and Film [25].

Avatars were controlled based on six-coordinate data (three positions and three rotations) obtained from the HMD and two controllers. The avatars had minimal personality traits to avoid confounding social cues, and their movements were implemented using the Final IK (Inverse Kinematics) asset. In the VR space, avatars faced each other, and an object appeared at the center between them (Figure 1). Reaction times were acquired via collision detection when avatars touched target objects. The objects used for the category judgment task consisted of 12 stimulus items, with three objects assigned to each combination of category (Kitchen/Garage) and difficulty level (Easy/Hard) (see Figure 2). Each object was presented with the handle positioned either on the left or the right side, resulting in a total of 24 target stimuli that were randomly presented during the experiment.

Based on a preliminary survey, difficulty levels were classified as Low (difficulty = 0) or High (difficulty = 1); each block of 24 trials contained 12 High-difficulty objects (Figure 2b). Objects were operationally defined as “more complex,” “Hard,” or “ambiguous” when they achieved categorization accuracy rates below 0.80 in preliminary testing, indicating substantial uncertainty in category membership. These high-difficulty objects included ambiguous items such as camping equipment that required collaborative judgment for accurate categorization. “Collaborative judgment” refers to trials where both participants needed to interact to resolve category uncertainty, as the objects’ ambiguous nature made individual categorization unreliable.

### 2.4. Experimental Design

**Figure 3:**
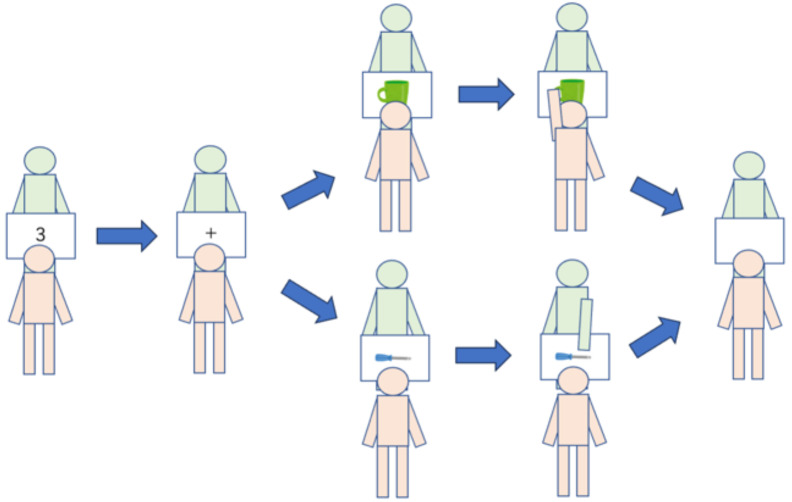
Experimental session flow diagram showing the progression from individual practice through collaborative sessions. *Note:* Participants move through four distinct phases: Practice (no category restriction), Individual (category-specific responses), Bot Pair (collaboration with bot), and Human Pair (human-human collaboration with role reversal).

The experiment consisted of four types of sessions (see Table 1 and Figure 3). The practice session contained eight trials; all other sessions contained 24 trials. Session types included: (1) Practice: Task familiarization with no category restriction, (2) Individual: Respond only to assigned category objects with partner present but no interaction, (3) Bot Pair: Paired with bot making opposite-category responses (always incorrect for High-difficulty objects), and (4) Human Pair: Paired with a human partner with category assignments swapped between partners.

**Design Rationale for Fixed Session Order:** The fixed order (Bot condition always preceding Human condition) was implemented to control category ambiguity prior to Session 4, which constitutes the primary experimental objective. By having the bot systematically provide incorrect category judgments for all high-difficulty (ambiguous) objects in Session 3, we induced a controlled level of category ambiguity across all participants. The research objective was to observe in Session 4 (Human Pair) the collaborative convergence process—how human pairs achieve shared cognition regarding initially ambiguous category concepts.

All target objects selected in the preliminary experiment were used in the main experiment. The presentation order of the objects was randomized; however, the sequence was controlled such that each category (Kitchen and Garage) and difficulty level (Easy and Hard) appeared an equal number of times. Within each session, all participants were presented with the same set of target objects. Each session consisted of 24 trials corresponding to the 24 target stimuli (12 unique objects × 2 handle orientations).

### 2.5. Measures

Behavioral data included: touch responses (1 = participant touch, −1 = partner touch, 0 = no touch), correctness (1 = correct, 0 = incorrect), reaction time (maximum 3 s), and difficulty level (High/Low). Eye movement synchrony data were collected using DTW analysis of eye orientation velocity components (x, y, z axes) for both object presentation periods and pre-object waiting periods.

### 2.6. Synchrony Analysis

In this study, gaze synchrony was analyzed based on pupil position data obtained from the HTC VIVE Pro Eye head-mounted display. Although the device also provides an estimated eye direction vector, this output frequently contained missing values and was therefore not suitable for reliable trial-level analysis. Instead, we directly treated the three-dimensional pupil position signals as gaze data. Sensor data were sampled at a variable rate (average 80 Hz). After removing missing values, client and host participant data were temporally synchronized and linked at approximately 0.02 s intervals. This approach allowed us to capture participants’ visual dynamics without depending on device-specific estimation procedures.

From the raw pupil position time series, we computed gaze orientation velocity components along the x, y, and z axes from temporal differences between successive samples. These velocity values represent instantaneous changes in eye orientation and were used as the basis for synchrony analysis. For each trial, paired time-series data from the two participants were compared using two complementary methods: (1) cross-correlation coefficients, which measure linear temporal alignment, and (2) Dynamic Time Warping (DTW) distances, which capture flexible similarity by allowing temporal shifts in gaze trajectories.

Because DTW distance is inversely related to similarity, the values were inverted and normalized to the [0, 1] range, resulting in a synchrony index where higher values indicate stronger gaze coordination. This normalized synchrony index was subsequently used as a parameter in the active inference model to examine its influence on collaborative decision-making.

**Figure 4:**
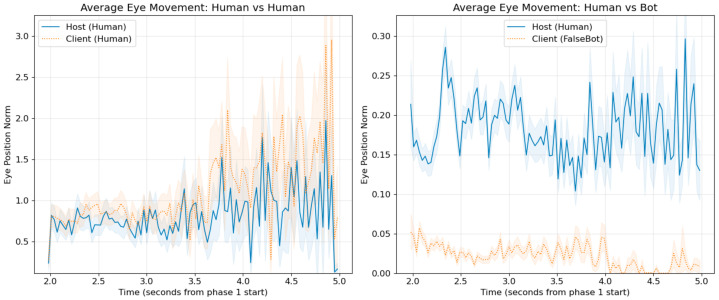
Time-series trajectories of three-dimensional pupil position during object presentation (approximately 3 s per trial) for Human–Human (**left**) and Human–Bot (**right**) conditions. The three subplots in each panel show the X, Y, and Z components of pupil position (arbitrary units from HTC VIVE Pro Eye sensor) averaged across all trials and participant pairs (N = 7 pairs for each condition). Blue solid lines represent host participants, orange dotted lines represent client participants or bot. Temporal synchrony is indicated by the degree of overlap and similar trajectory patterns between the two lines. The (**left**) demonstrates stronger gaze coordination in Human–Human pairs, with closer temporal alignment and similar movement patterns across all three spatial dimensions, compared to the more divergent patterns in Human–Bot interactions (**right**).

Figure 4 left shows the time-series averages of gaze trajectories for Human–Human pairs, while the right panel shows those for Human–Bot pairs. The Human–Human pairs exhibit stronger synchrony overall, although it is also evident that the human partner’s pupil dynamics are modulated when interacting with a Bot, producing patterns distinct from Human–Human interactions. It should be noted that these graphs represent averaged trajectories; actual DTW-based synchrony values vary across individual pairs.

### 2.7. Model–Data Loop (Overview of Figure 5)

**Figure 5:**
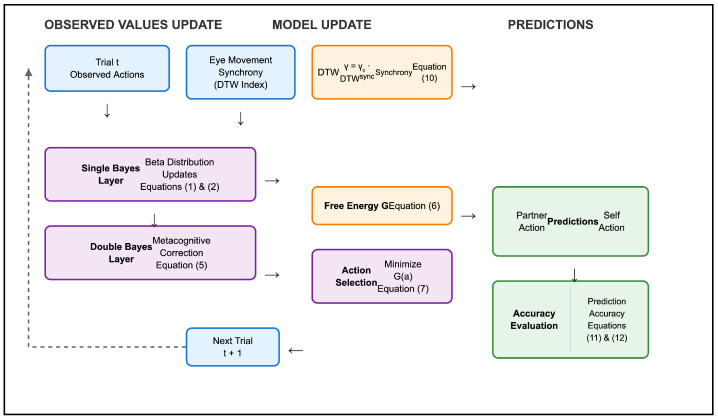
Active inference model with eye-gaze synchrony. *Note:* The model integrates a two-layer Bayesian update (Single Bayes, Double Bayes) with free energy minimization. Partner prediction and self-action selection are modulated by a synchrony parameter (γ), derived from eye-movement synchrony (DTW), to enhance collaborative agreement.

Figure 5 illustrates the procedure linking empirical data with the active inference model. At each trial, observed values—self and partner actions together with gaze synchrony indices—update the lower-level Bayesian layer. These beliefs are then passed to the higher-level Bayesian layer, which refines predictions of partner behavior. In parallel, the synchrony index is converted into the γ parameter that modulates collaborative weighting.

Based on these updated beliefs, the model evaluates candidate self-actions using the free-energy function, and a softmax policy determines the next action probability. From this process, both partner-action predictions and self-action policies are derived and compared with the subsequent trial’s observed data. Accuracy and free-energy trajectories are then plotted across trials, focusing on the 12 high-difficulty objects.

The mathematical details of Bayesian updating, synchrony weighting, and free-energy minimization are provided in the Section 3.

### 2.8. Bot Avatar Gaze Control System Implementation

Bot gaze behavior was implemented to simulate joint attention with human partners. The system used VRM (Virtual Reality Model) LookAtHead components with gaze targets dynamically updated based on experimental context. During pre-object and post-object waiting periods, the bot tracked a transparent target object positioned at the endpoint of the human partner’s gaze direction, enabling the bot to follow the partner’s visual attention in real-time. During object presentation, gaze direction shifted to experimental objects. All gaze transitions utilized integrated smoothing (factor 5.0) with gaze vectors projected 2.0 m from the avatar’s head for natural eye movements. The resulting gaze orientation and position data were transmitted through the same network protocol as human eye-tracking data for synchronized recording.

## 3. Results

### 3.1. Manipulation Check

**Figure 6:**
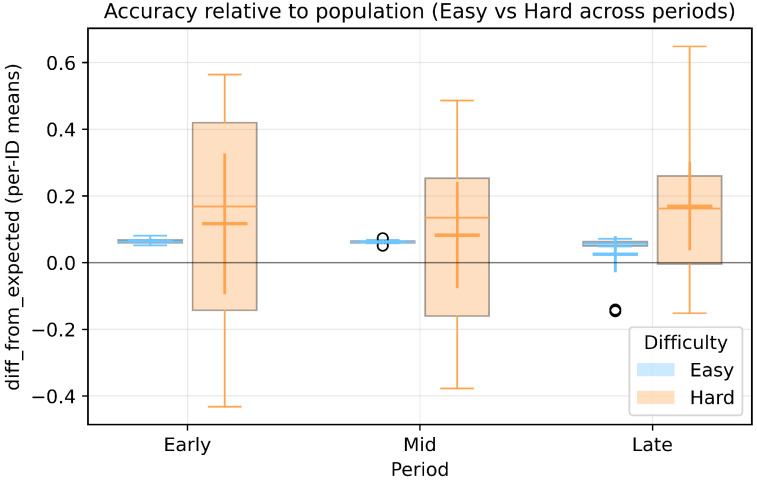
Changes in response accuracy across periods by task difficulty (Easy/Hard) in the Human condition. *Note:* Each period consists of 8 trials out of a total of 24 trials, and the plots are based on per-ID means. Boxplots show the “difference from population mean” (diff_from_expected) with 95% confidence intervals of the means. Results demonstrate category concept convergence in Hard trials and stability in Easy trials.

As a manipulation check, we tested whether task difficulty influenced response accuracy (measured as the deviation from the general population’s accuracy, given that the task included ambiguous categories such as camping gear). No significant effect of difficulty was found on the mean response accuracy, whereas a significant effect was observed on the variance (Figure 6).

To further examine variance differences, a two-way Brown–Forsythe ANOVA on absolute deviations from group medians showed a significant main effect of difficulty on response variance (F(1,76) = 61.06, *p* < 0.001), no significant main effect of period (F(2,76) = 1.12, *p* = 0.331), and a marginally significant difficulty × period interaction (F(2,76) = 2.58, *p* = 0.082).

These manipulation check results confirmed that responses to difficult tasks showed high variance, validating that our experimental conditions successfully induced the ambiguous category concepts assumed in this study. Although the interaction effect was not statistically significant (*p* = 0.082), visual inspection of the data suggested a descriptive pattern of variance convergence toward later trials.

### 3.2. Eye Movement Synchrony Analysis

We analyzed eye movement synchrony between participants using sensor data from the VR headsets during object presentation. Two complementary measures were employed to quantify collaborative gaze behavior: (1) Dynamic Time Warping (DTW) synchrony indices, which evaluate shape similarity across time-series trajectories, and (2) cross-correlation coefficients, which capture instantaneous coordination of eye movement vectors. Within-subjects comparisons were conducted across conditions for each participant.

For **DTW synchrony**, we applied a mixed linear model with logit-transformed synchrony values as the dependent variable and participant ID as a random effect. Results revealed that Human–Human pairs exhibited significantly higher synchrony compared to Human–Bot pairs (MixedLM: condition effect = −0.668, SE = 0.057, z = −11.66, *p* < 0.001). The estimated marginal means were Human = 0.440, FalseBot = 0.605, with a difference of 0.165 on the 0–1 scale. Post hoc power analysis using G*Power version 3.1.9.6 (generic z-test) confirmed adequate statistical power for this effect (noncentrality parameter μ = 0.668, noncentral distribution SD σ = 0.057, α = 0.05, two-tailed; critical z = 1.96, power = 1.00).

For **instantaneous gaze coordination measured by cross-correlation**, trial-level values were averaged per individual, and a paired Wilcoxon signed-rank test was conducted between matched participants across conditions. Human–Human pairs demonstrated significantly higher instantaneous coordination than Human–Bot pairs (paired Wilcoxon: W = 10.00, *p* = 0.005, Cohen’s d = 0.85). Mean cross-correlations were Human = 0.048 (SD = 0.058) and FalseBot = −0.005 (SD = 0.050).

Both indices converge in demonstrating that human–human interactions generate more coordinated gaze patterns compared to human–bot interactions. Crucially, DTW synchrony reflects shape similarity across entire time-series trajectories, whereas cross-correlation captures instantaneous directional coordination. These complementary measures address distinct facets of gaze synchrony in collaborative contexts.

### 3.3. Active Inference Model Results

To evaluate participants’ partner behavior prediction accuracy and self-behavior prediction accuracy in the pair task, we implemented and compared Bayesian learning with active inference models.

#### 3.3.1. Active Inference Model Architecture

The model explored in this study aims to capture the process of sharing ambiguous category concepts by predicting others’ internal models. Therefore, we attempted to construct an active inference model that learns sequentially from observed values and can predict partner behavior more accurately.

We calculated KL divergence from the difference between the “probability distribution from the previous trial (prior distribution)” and the “updated probability distribution of the current trial (posterior distribution)”, and compared a model that weights the synchrony parameter gamma to the KL term with a model that weights it to the reward (risk) term. As a result, both models showed roughly equivalent partner prediction accuracy of Precision = 1.000, Recall = 0.202, F1 = 0.336, AUC = 0.652, MCC = 0.378, while the latter showed Precision = 0.958, Recall = 0.404, F1 = 0.568, AUC = 0.769, MCC = 0.534, but, a tendency for accuracy to remain unchanged as trials progressed, particularly decreasing in the former, was confirmed. Next, we redefined the KL definition based on Equation (7) as the difference between “probability distribution after simple Bayes update” and “probability distribution after double Bayes update”, and compared models where gamma is applied to the KL term, applied to the reward term, and mixed models. As a result, the model applying gamma to the KL term did not improve the learning curve, and only the model applying gamma to the reward term showed the most stable accuracy improvement throughout trials. The latter showed the highest performance with model accuracy throughout trials of Precision = 0.964, Recall = 0.465, F1 = 0.627, AUC = 0.831, MCC = 0.583, so this model was finally adopted.

The adopted model extends the active inference framework to capture socially shared cognition through two theoretical innovations. First, we redefine the KL divergence term to represent the discrepancy between first-order partner prediction (simple Bayes) and second-order belief updating (double Bayes). This dual-layer architecture captures how individuals not only predict others’ actions but also iteratively refine those predictions through social interaction. Second, the collaborative weighting parameter γ (representing gaze synchrony) modulates the reward term rather than the KL term, reflecting how embodied synchrony influences agreement-seeking behavior. As demonstrated in the model comparison above, this formulation achieved superior fit to observed data compared to standard single-layer models or alternative γ-weighting schemes.

Our model implements an active inference framework based on dual-layer Bayesian inference that integrates eye-gaze synchrony for collaborative decision-making. The framework employs two hierarchical layers: (1) Single Bayes provides direct partner behavior prediction $P(A_{p}=1|A_{s},O)$, and (2) Double Bayes applies iterative belief refinement yielding $Q(A_{p}=1|A_{s},O)$.

Bayesian Update Process: At trial *t*, the first layer maintains Beta distributions for partner predictions:

(1) $$P_{t}\left(A_{p}=1|A_{s}=1,O\right)∼\mathrm{Beta}\left(\alpha_{11},\beta_{11}\right)$$

(2) $$P_{t}\left(A_{p}=1|A_{s}=0,O\right)∼\mathrm{Beta}\left(\alpha_{01},\beta_{01}\right)$$

where $A_{s}\in \left\{0,1\right\}$ represents the self action (1 = touch object, 0 = no touch), $A_{p}\in \left\{0,1\right\}$ represents the partner action with the same encoding, and *O* represents the object.

Upon observing actions $\left(a_{s}^{t-1},a_{p}^{t-1}\right)$, parameters update as:

(3) $$\alpha_{ij}\leftarrow \alpha_{ij}+\delta \left(a_{s}^{t-1}=i,a_{p}^{t-1}=j\right)$$

(4) $$\beta_{ij}\leftarrow \beta_{ij}+\delta \left(a_{s}^{t-1}=i,a_{p}^{t-1}\neq j\right)$$

where $a_{s}^{t-1},a_{p}^{t-1}$ are the observed actions from the previous trial (lowercase denotes observed values, superscript t−1 indicates the previous trial), and δ(·) is the Kronecker delta function (equals 1 when the condition is true, 0 otherwise). The Beta distribution parameters $\alpha_{ij},\beta_{ij}$ are maintained for each combination of $i=A_{s},j=A_{p}$.

The second layer employs exponential moving averages for double Bayesian updating:

(5) $$Q_{t}\left(A_{p}=1|A_{s}=k\right)=\alpha \cdot a_{p}^{t-1}+\left(1-\alpha \right)\cdot Q_{t-1}\left(A_{p}=1|A_{s}=k\right)$$

when $a_{s}^{t-1}=k$, where $P(A_{p}=1|A_{s},O)$ represents the single Bayes prior distribution for predicting partner behavior based on self action and object, while $Q(A_{p}=1|A_{s},O)$ represents the double Bayes posterior distribution incorporating iterative belief refinement.

Free Energy Calculation: For each candidate action a∈{0,1}, free energy is defined as:

(6) $$G_{t}^{\left(a\right)}=\kappa \frac{D_{\mathrm{KL}}\left(\mathrm{Bern}\left(q_{t}^{\left(a\right)}\right)∥\mathrm{Bern}\left(p_{t}^{\left(a\right)}\right)\right)}{1+D_{\mathrm{KL}}\left(\mathrm{Bern}\left(q_{t}^{\left(a\right)}\right)∥\mathrm{Bern}\left(p_{t}^{\left(a\right)}\right)\right)}+\lambda_{0}\gamma_{t}\left(2q_{t}^{\left(a\right)}-1\right)$$

where $G_{t}^{\left(a\right)}$ is the free energy for self action a∈{0,1} at trial *t* (lower values indicate preferred actions), κ is the scaling parameter for the KL divergence term (set to 0.1), $D_{\mathrm{KL}}\left(\mathrm{Bern}\left(q\right)∥\mathrm{Bern}\left(p\right)\right)$ is the Kullback–Leibler divergence between Bernoulli distributions with parameters *q* and *p*, $q_{t}^{\left(a\right)}=Q_{t}\left(A_{p}=1|A_{s}=a\right)$ is the predicted probability of partner action given self action *a* from the double Bayes layer, $p_{t}^{\left(a\right)}=P_{t}\left(A_{p}=1|A_{s}=a\right)$ is the predicted probability from the single Bayes layer, $\lambda_{0}$ is the base reward weighting parameter, $\gamma_{t}$ is the gaze synchrony weight at trial *t* (derived from DTW analysis), and $R\left(a\right)\equiv \left(2q_{t}^{\left(a\right)}-1\right)$ is the collaborative agreement reward term.

In the present model, gaze synchrony is not treated as an outcome variable indicating whether agreement has been achieved. Rather, gaze synchrony is regarded as an index reflecting largely unconscious processes of mutual attention, and is used as a weighting parameter for the cooperative agreement term—that is, how strongly aligning one’s action with the partner is emphasized during inference.

Accordingly, gaze synchrony does not represent the result of agreement itself, but modulates the extent to which social information is incorporated into the inferential process leading toward agreement.

The KL divergence for binary distributions is computed as:

(7) $$D_{\mathrm{KL}}\left(\mathrm{Bern}\left(q\right)∥\mathrm{Bern}\left(p\right)\right)=q\log\frac{q}{p}+\left(1-q\right)\log\frac{1-q}{1-p}$$

where numerical stability is ensured through epsilon clipping ($\epsilon =10^{-12}$) to prevent logarithmic singularities. The compression term $\frac{D_{\mathrm{KL}}}{1+D_{\mathrm{KL}}}$ maps the KL divergence to the range [0,1) for balanced weighting with the reward term. The second term $\left(2q_{t}^{\left(a\right)}-1\right)$ represents a risk component that increases when disagreement with the partner’s category judgment is expected. This serves as a target term that encourages action selection toward anticipated agreement.

Action Selection: Action selection follows the softmax function:

(8) $$a_{t}=\arg\min_{a}G_{t}^{\left(a\right)},\;\pi_{t}\left(a\right)=\frac{\exp\{-G_{t}^{\left(a\right)}/\tau \}}{\sum_{a^{\prime }}\exp\left\{-G_{t}^{\left(a^{\prime }\right)}/\tau \right\}}$$

where $a_{t}$ is the selected action at trial *t* (deterministic selection via free energy minimization), $\pi_{t}\left(a\right)$ is the probability of selecting action *a* at trial *t* (computed for analysis purposes), and τ is the softmax temperature parameter controlling the randomness in action selection (lower values increase deterministic behavior). The actual action selection uses argmin deterministically, while $\pi_{t}\left(a\right)$ provides the selection probabilities for model evaluation.

Model Parameters: The model employs the following parameter settings: (1) The softmax temperature parameter τ=1.0 balances exploration and exploitation in action selection. (2) The exponential moving average (EMA) decay rate α=0.8 determines the weight given to previous observations in double Bayesian belief updates, balancing recent information with historical context. (3) Beta distribution priors for category belief states were initialized with uniform priors: $\alpha_{ij}=1,\beta_{ij}=1$ for all action combinations, representing maximum uncertainty before any observations. (4) The base reward weight $\lambda_{0}=1.0$ scales the collaborative agreement term in the free energy calculation, maintaining balanced contributions from information gain and social coordination. (5) The gaze synchrony weight $\gamma_{0}\in \left\{0,0.1,0.5,0.9\right\}$ modulates the influence of embodied synchrony on collaborative decision-making.

#### 3.3.2. Synchrony Parameter Integration

DTW Synchrony Calculation: Eye movement synchrony is calculated using Dynamic Time Warping (DTW) distance between participants’ eye orientation velocity components. The DTW distance values are normalized to create a synchrony index:

(9) $${\mathrm{DTW}}_{\mathrm{sync}}^{\mathrm{raw}}=-{\mathrm{DTW}}_{\mathrm{distance}}$$

(10) $${\mathrm{DTW}}_{\mathrm{sync}}^{\mathrm{normalized}}=\frac{{\mathrm{DTW}}_{\mathrm{sync}}^{\mathrm{raw}}-\min\left({\mathrm{DTW}}_{\mathrm{sync}}^{\mathrm{raw}}\right)}{\max\left({\mathrm{DTW}}_{\mathrm{sync}}^{\mathrm{raw}}\right)-\min\left({\mathrm{DTW}}_{\mathrm{sync}}^{\mathrm{raw}}\right)}$$

where ${\mathrm{DTW}}_{\mathrm{raw}}$ is the raw DTW distance (negative values) and ${\mathrm{DTW}}_{\mathrm{norm}}$ is the normalized DTW synchrony index scaled to [0, 1] range, with higher values indicating greater synchrony.

Synchrony-Weighted Collaboration: The synchrony parameter modulates collaborative decision-making:

(11) $$\gamma =\gamma_{0}\cdot {\mathrm{DTW}}_{\mathrm{sync}}^{\mathrm{normalized}}$$

where γ is the synchrony weight acting as a weighting factor for social coordination in self action selection, and $\gamma_{0}\in \left\{0,0.1,0.5,0.9\right\}$ represents the base synchrony weight parameter (baseline values for setting coordination importance). Higher synchrony values enhance collaborative agreement weighting in the γ·R(a) term of Equation (6). Model Evaluation: Model performance is assessed through prediction accuracy measures:

(12) $${\mathrm{Acc}}_{s}=E\left[1\left(a_{s}^{*}=a_{s}^{\mathrm{true}}\right)\right]$$

(13) $${\mathrm{Acc}}_{p}=E\left[1\left(1\left(\hat{P}\left(A_{p}=1\right)\geq 0.5\right)=a_{p}^{\mathrm{true}}\right)\right]$$

where ${\mathrm{Acc}}_{s}$ is the self-action prediction accuracy (proportion of correctly predicted self actions), ${\mathrm{Acc}}_{p}$ is the partner action prediction accuracy (proportion of correctly predicted partner actions), and FE is the minimum free energy value calculated as FE=min(G(0),G(1)) to evaluate action preferences.

#### 3.3.3. Comparative Performance Analysis

**Figure 7:**
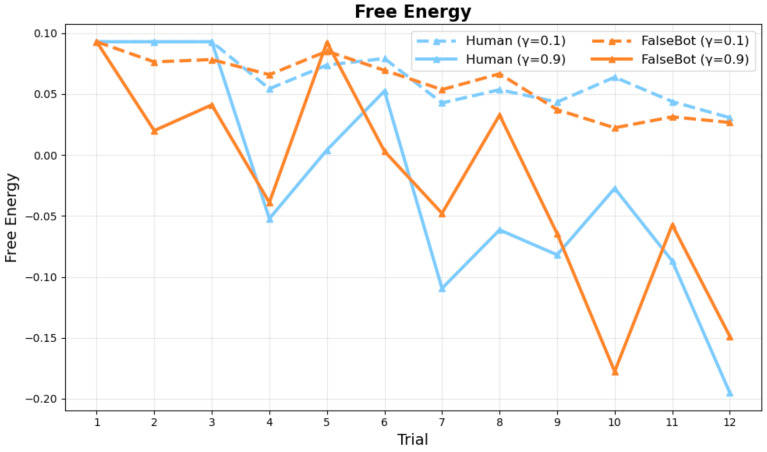
Changes in Free Energy Across Session Progression. *Note:* G values based on active inference updated with observed values from each trial. Free energy decreases over trials, indicating progress in internal model updating. Higher gaze synchrony parameters (e.g., $\gamma_{0}=0.9$) show overall lower free energy values than lower parameters (e.g., $\gamma_{0}=0.1$). Bot condition shows more linear changes due to consistent bot behavior.

Free energy decreases across trials, indicating that internal model updating is progressing. Higher gaze synchrony parameters show overall lower free energy values than lower parameters. The Bot condition shows more linear changes because bot behavior is consistent (Figure 7).

**Figure 8:**
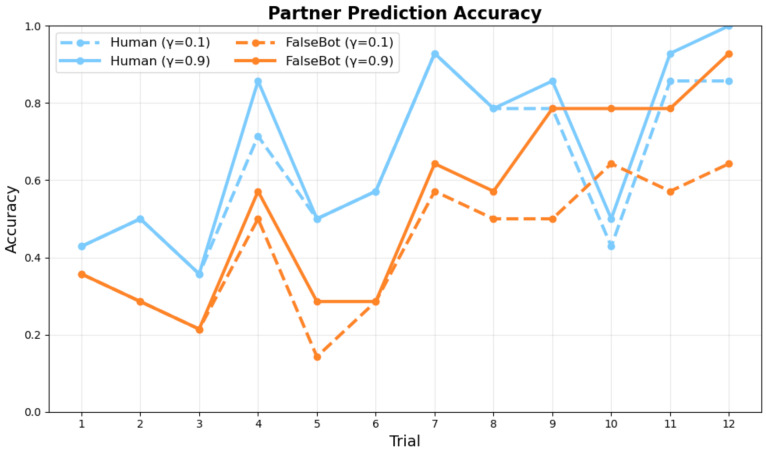
Partner Prediction Accuracy across trials.

**Figure 9:**
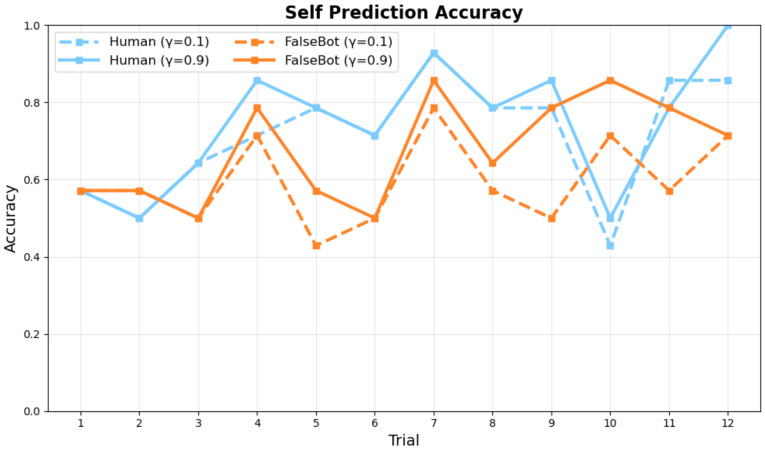
Self Prediction Accuracy across trials.

Figure 8 and Figure 9 show the comparative performance between conditions with and without synchrony weighting in the active inference model across trials for ambiguous category judgment objects.

The results demonstrate that synchrony weighting significantly enhances prediction accuracy, particularly for Human pairs. In the Human condition, synchrony weighting improved both partner prediction accuracy and self-behavior prediction accuracy. Bot pairs showed more modest improvements with synchrony weighting, consistent with reduced interpersonal coordination in human-bot interactions.

Table 2 presents the comprehensive accuracy comparison across synchrony weight conditions. When comparing overall accuracy across all trials, synchrony weighting at $\gamma_{0}=0.1$, $\gamma_{0}=0.5$, and $\gamma_{0}=0.9$ showed notable performance differences compared to the baseline condition ($\gamma_{0}=0$). The results indicate that synchrony contributes significantly to collaborative agreement as a reward mechanism, while $\gamma_{0}=0.5$ and $\gamma_{0}=0.9$ yielded optimal and equivalent performance for Human pairs, this suggests that synchrony weighting reaches saturation around $\gamma_{0}=0.5$ for Human pairs, though Bot pairs show continued improvement up to $\gamma_{0}=0.9$.

Figure 8 and Figure 9 show the model’s prediction accuracy for partner and self actions, respectively, across trials. Figure 8 shows how accurately the active inference model predicted the partner’s next action at each trial. Human pairs (blue lines) demonstrate superior learning trajectories compared to Bot pairs (orange lines) across different synchrony weighting conditions ($\gamma_{0}$ = 0.1 and $\gamma_{0}$ = 0.9). Figure 9 shows how well the model predicted participants’ actual behavior (i.e., agreement between model predictions and observed participant actions). Both measures approach 1.0 by the final trial, indicating successful learning. The results demonstrate clear learning trajectories, with final trial (Trial 12) accuracies reaching near-optimal values under high synchrony conditions. Specifically, at $\gamma_{0}=0.5$–0.9, Human pairs achieved partner prediction accuracy of 0.93 and self prediction accuracy of 1.0 by Trial 12, demonstrating successful collaborative concept convergence. Partner action prediction accuracy increases more rapidly than self-action prediction accuracy, reflecting the model’s progressive understanding of collaborative dynamics. Across both measures, Human pairs show higher prediction accuracy than Bot pairs, particularly in early and middle trials.

#### 3.3.4. Final Trial Performance Analysis

To examine learning convergence, we analyzed prediction accuracy specifically for the final trial (Trial 12), representing the endpoint of collaborative concept learning (Table 3).

Results demonstrate substantial improvement in final trial performance under high synchrony weighting conditions. In the Human condition with $\gamma_{0}=0.5$–0.9, self-prediction accuracy reached perfect performance (1.0), while partner prediction accuracy achieved 0.93. This indicates that incorporating gaze synchrony as a collaborative weighting parameter enables the model to capture the convergence toward shared category concepts that occurs through 12 trials of joint learning.

The Bot condition showed more modest improvements (partner accuracy 0.86, self accuracy 0.79 at $\gamma_{0}=0.9$), consistent with reduced opportunities for genuine interpersonal coordination when one partner exhibits non-adaptive, predetermined behavior patterns. Notably, $\gamma_{0}=0.0$ and $\gamma_{0}=0.1$ showed identical performance, confirming that the model requires non-zero synchrony weighting to benefit from gaze coordination information.

## 4. Discussion

This study attempted to apply active inference models to the process of collaborative learning between pairs, specifically the agreement process for ambiguous category concepts. We performed beta distribution updates from observed values, followed by double Bayesian updating to predict partner internal models, and calculated free energy from active inference models based on these results, attempting to match model predictions with experimental observations.

Experimental results showed that our model demonstrated progressive learning across trials, ultimately achieving high accuracy in predicting both partner and self next actions. Additionally, when partner category concept agreement was added as a reward term to the G equation, with the gaze synchrony index DTW set as a parameter representing attention to partner attention, higher gaze synchrony resulted in improved model accuracy, with optimal performance achieved at synchrony weights of $\gamma_{0}=0.5$–0.9. At these levels, the model successfully captured the collaborative convergence process, achieving near-perfect self-prediction (1.0) and high partner-prediction accuracy (0.93) by the final trial in Human pairs.

The handle task situation in this study, namely the task of reaching out to objects as collaborative work with others facing each other, has also been used in studies of imitation behavior [26]. The underlying body movement and attention synchrony for concept sharing is also related to triadic relationships known in developmental research of interpersonal communication. The results of this study, obtained from task situations with such psychological breadth, are expected to provide clues for approaching the symbol grounding problem in concept acquisition in AI research by utilizing body movements and attention synchrony.

A methodological contribution of this study lies in the integration of dynamic time warping (DTW) synchrony indices into the active inference framework as a γ–weighting parameter, while DTW has been widely used in cognitive science to quantify temporal alignment of behavioral signals, its application as a modulator of the free energy functional is novel. By treating gaze synchrony as a factor influencing the balance between risk and information gain, the present approach extends active inference beyond purely internal predictive mechanisms toward an embodied, interaction-sensitive formulation. From this perspective, gaze synchrony is not an explicit goal optimized by the agents. Instead, it functions as a contextual factor reflecting an unconscious orientation toward coordination, which in turn influences how strongly agreement-related information is weighted during social inference.

This multimodal integration of gaze sensing, motion sensing (touch behavior), and cognitive process modeling addresses a critical gap in embodied AI research. As physical AI and humanoid robots increasingly enter collaborative settings with humans, understanding how lower-level sensorimotor synchrony (gaze, gesture) contributes to higher-level cognitive alignment (shared concepts, mutual understanding) becomes essential. Our framework demonstrates that computational models of human–robot collaboration must account for the dynamic interplay between observable behavioral signals and latent cognitive states—a principle that applies broadly to designing robots capable of natural, adaptive interaction with human partners.

### Comparison to Existing HRI Frameworks

Compared to reinforcement learning or imitation learning approaches in HRI, active inference offers advantages for modeling collaborative convergence in ambiguous situations. Unlike reinforcement learning requiring explicit reward functions or imitation learning limited to observed trajectories, active inference naturally captures uncertainty through free energy minimization. The dual-layer architecture enables reasoning about partner beliefs—essential for theory-of-mind computations in social robots but absent from standard approaches.

## 5. Limitations

This study has several limitations that should be acknowledged. First, the sample size was modest (N = 14 participants, 7 pairs), which limits statistical power for detecting subtle effects and generalizability of findings. Second, the experimental setting focused on a specific category learning task in controlled VR environments, which may not fully generalize to more complex real-world collaborative scenarios. Third, the Bot agents exhibited relatively simple and predetermined gaze behaviors, limiting the ecological validity of human-Bot interactions. More sophisticated bidirectional models, where both partners engage in active inference processes simultaneously, would allow for richer simulations of mutual adaptation and negotiation in joint tasks.

Fourth, in this study, comparisons of gaze synchrony between bot pairs and human pairs were conducted under the constraint that the order from Session 3 to Session 4 was fixed. Because this design does not incorporate counterbalancing, potential order effects—such as task familiarity or strategy adaptation across sessions—cannot be fully ruled out. Accordingly, differences in gaze synchrony across sessions should be interpreted as exploratory rather than as definitive evidence of causal effects, while these potential confounds are inherent to our theoretical question about collaborative convergence following induced uncertainty, future studies could complement this approach by examining collaborative dynamics without induced ambiguity or implementing wash-out periods between conditions to minimize transfer effects.

Fifth, the current implementation processes data offline. Real-time deployment in physical HRI systems would require computational optimization, particularly for embedded robot platforms. Future work should investigate lightweight implementations such as amortized inference or parallelized filtering suitable for real-time robot control (10–30 Hz), along with online learning mechanisms for individual user adaptation.

Sixth, the relatively small sample size (N = 14 participants, 7 pairs) and exploratory nature of some analyses mean that overfitting cannot be entirely ruled out, while the model achieved high prediction accuracy on the observed data, this should be interpreted cautiously. The primary aim of this study was to provide a proof of concept demonstrating how active inference can model collaborative convergence in virtual reality, rather than to establish definitive generalization performance. Future work should validate the proposed framework using larger datasets and independent samples to assess its broader applicability.

## 6. Conclusions

This study demonstrated that active inference models incorporating gaze synchrony can effectively predict collaborative decision-making in VR environments. The theoretical contribution lies in extending active inference to model socially shared cognition through embodied multimodal synchrony. By treating gaze coordination as a factor modulating free energy minimization, we provided a computational framework that bridges individual predictive processing and interpersonal alignment. The integration of DTW synchrony indices into the active inference framework offers a novel approach to quantifying and modeling collaborative agreement in joint action scenarios.

From a practical perspective, this approach provides insights for designing socially intelligent agents in human–robot interaction contexts. Applications include educational robotics (tutoring robots inferring student understanding), therapeutic robots for autism (adapting to atypical gaze patterns), collaborative assembly robots (predicting worker intentions), and elderly care robots (detecting confusion through synchrony breakdown). Beyond robotics, VR-based training environments could provide real-time feedback on collaboration quality in surgical training, aviation, or emergency response contexts.

Future work should examine more complex collaborative tasks, larger participant samples, and bidirectional active inference models where both partners simultaneously engage in mutual prediction. Extending this framework to other multimodal synchrony measures (gesture, speech rhythm) could further enrich our understanding of socially shared cognition. The present findings establish a foundation for computational modeling of shared understanding through embodied interaction and multimodal sensing in virtual environments.

## Figures and Tables

**Table 1:** Experimental Session Types and Descriptions.

Session	Name	Description
1	Practice	Task familiarization, no category restriction.
2	Individual	Respond only to assigned category objects (right hand only). Partner present but no interaction.
3	Bot Pair	Paired with bot. Bot always responds correctly to Easy objects but always responds incorrectly to Hard objects.
4	Human Pair	Paired with a human partner; category assignments swapped between partners.

**Table 2:** Model Accuracy Comparison Across Synchrony Weight Conditions.

Sync Weight	Condition	Partner Acc	Self Acc
0.0	Human	0.5833	0.7262
	Bot	0.4345	0.5952
0.1	Human	0.5833	0.7262
	Bot	0.4345	0.5952
0.5	Human	0.6190	0.7619
	Bot	0.5357	0.6726
0.9	Human	0.6190	0.7619
	Bot	0.5417	0.6786

**Table 3:** Prediction Accuracy at Final Trial (Trial 12) by Synchrony Weight.

Sync Weight	Condition	Partner Acc	Self Acc
0.0	Human	0.7857	0.8571
	Bot	0.6429	0.7143
0.1	Human	0.7857	0.8571
	Bot	0.6429	0.7143
0.5	Human	0.9286	1.0000
	Bot	0.8571	0.7857
0.9	Human	0.9286	1.0000
	Bot	0.8571	0.7857

## Data Availability

The data supporting the findings of this study, excluding biometric information, are available from the Open Science Framework at https://osf.io/7u4yz/files, accessed on 1 December 2025.

## Abbreviations

The following abbreviations are used in this manuscript:

VR	Virtual Reality
HRI	Human-Robot Interaction
JA	Joint Attention
DTW	Dynamic Time Warping
KL	Kullback–Leibler
VA	Virtual Agent
HRC	Human-Robot Collaboration
GOS	Gaze Object Sequence
HMD	Head-Mounted Display
MCC	Matthews Correlation Coefficient
AUC	Area Under the Curve
